# Imatinib in chronic myeloid leukemia elderly patients

**DOI:** 10.18632/aging.100420

**Published:** 2011-12-27

**Authors:** Gabriele Gugliotta, Fausto Castagnetti, Francesca Palandri, Michele Baccarani, Gianantonio Rosti

**Affiliations:** Department of Hematology and Clinical Oncology “Seràgnoli”, S. Orsola-Malpighi Hospital, University of Bologna, Italy

The median age at diagnosis of Chronic Myeloid Leukemia (CML) reported in clinical trials is generally of 50-55 years; it is higher (more than 60 years) in epidemiologic registries and in observational studies [1]. Therefore, a significant proportion of CML patients are “elderly”, according to the most widely accepted definition of “old person” (age > 65 years).

Prior to imatinib (IM) introduction, an orally taken tyrosine kinase inhibitor, older age was associated to a worse outcome (lower response rates and lower long-term survival), regardless of the treatment used (busulfan, hydroxyurea, interferon alpha), basically, for unknown reasons. It can be speculated that, with interferon, the reason of an inferior outcome of elderly patients lied in a poorer compliance; however, this worsen prognosis of elderly CML patients have been described even employing well tolerated drugs (like hydroxyurea). Age was included in the 2 most used risk scores for CML: Sokal score (1984), referring to patients treated with conventional chemotherapy, and EURO score (1998), for patients treated with interferon [2, 3].

The introduction of IM has revolutionized the therapy of CML patients, significantly improving survival [4]. However, in the early days of IM, many physicians felt that the prognosis of an old CML patient, particularly those older than 75-80 yrs, could not be positively influenced by IM and a not negligible part of elderly patients are still not allocated to IM (or enrolled in clinical trials) for different reasons (co-morbidities, toxicity, long term outcome, socio-economic factors) [5].

The importance of age in the IM era has been investigated in different studies, leading to a re-evaluation of its prognostic significance. A large study in late chronic phase patients treated with IM after interferon failure, conducted by GIMEMA (Gruppo Italiano Malattie Ematologiche dell'Adulto) CML Working-Party (WP), showed lower hematologic and cytogenetic response rates in older patients (> 65 years old), but similar overall survival with a 36 months median follow-up [6]. In another large but heterogeneous (early chronic phase, late chronic phase, and advanced phases) series of patients treated with IM at the MDACC, older age appeared to be not associated with a worse outcome; however, the cut-off to define older patients was 60 years, elderly early chronic phase patients were few (49) and no long term data were available [7].

The first study to evaluate the effect of age in large series of front-line IM treated patients and with a long follow-up was recently published by the GIMEMA CML WP [8]. Five hundred and fifty nine patients, of whom 115 ≥ 65 years old, were enrolled in 3 prospective, multicenter, trials. After a median follow-up of 60 months, hematologic, cytogenetic and molecular response rates were identical in older and younger patients while survival was significantly inferior in older patients. This lower survival was due to a higher number of deaths occurred in chronic phase, reasonably unrelated to IM therapy or toxicity, and not to a higher progression rate in older patients.

Recently a new prognostic score was elaborated based on 2060 patients enrolled in studies of first-line IM-based regimes and collected by the European Leukemia Net / EUTOS Registry [9]. Of the all variables analyzed, only spleen size and basophils percentage, best discriminated between high-risk and low-risk patients; age had no more a prognostic impact, a further confirmation that IM has cancelled the differences in prognosis between younger and older patients.

No doubt, the overall toxicity and the need for dose optimization are higher for IM with respect to conservative and palliative treatments, like hydroxyurea, particularly when older patients are treated; however, in the hands of expert physicians IM is generally manageable. Moreover, as the lower survival of elderly patients is mainly determined by age-related factors and co-morbidities, rather than by the progression of CML, a geriatric assessment (frailty status) may be helpful in the decision to allocate selected patients to IM therapy [10].

In conclusion, age had relevance when therapeutic strategies, like IM, were not available. However, today, older age *per se* must not be a limitation for treating patients with IM and the enrollment of these patients in clinical trials should be encouraged.

Second generation tyrosine kinase inhibitors (dasatinib and nilotinib) as front-line therapy showed higher response rates and lower toxicities compared to IM [11, 12]; probably these results will be confirmed also in elderly patients. Importantly, extra-hematologic toxicities are distinct between IM, dasatinib and nilotinib, allowing the selection of the more appropriate drug in relation to the presence of co-morbidities. Although data on dasatinib and nilotinib in elderly patients are still few and the follow-up is still short, these second generation tyrosine kinase inhibitors will probably further improve the outcome of CML elderly patients.
